# Can social media promote vaccination? Strategies and effectiveness of COVID-19 vaccine popularization on Chinese Weibo

**DOI:** 10.3389/fpubh.2024.1436632

**Published:** 2024-12-04

**Authors:** Jing Xu, Difan Guo, Jing Wu, Jinghong Xu

**Affiliations:** ^1^School of Journalism and Communication, Huaqiao University, Xiamen, China; ^2^School of Journalism and Communication, Beijing Normal University, Beijing, China; ^3^School of Arts and Communication, Beijing Normal University, Zhuhai, China; ^4^International College, Krirk University, Bangkok, Thailand

**Keywords:** vaccines, COVID-19, vaccine acceptance, sentiment, social media, outreach strategy

## Abstract

**Background:**

The COVID-19 pandemic has shown a high severity in terms of mortality, and to mitigate the impact of the COVID-19 pandemic, a great deal of reliance has been placed on vaccines with defensive effects. In the context of the transmission of hazardous Omicron variant strains, vaccine popularization and acceptance are very important to ensure world health security. Social media can spread information and increase public confidence in and acceptance of vaccines.

**Method:**

In this study, weibos related to “vaccine science popularization” during the COVID-19 pandemic in China were collected, and Weibo publishers were divided into Individuals, Organizations, Media, Government, and Scientists. The communication strategies were analyzed with content analysis from the four dimensions of Issue, Topic, Frame, and Position. SnowNLP was used to mine the audience comments and to assess their emotional tendencies. Finally, hierarchical regression was used to verify the causal relationship between vaccine science popularization strategies and audiences’ emotions.

**Results:**

We found that the higher the scientific authority of the weibo publisher, the more positive the emotional tendency of the audience toward the weibo. Issues that are scientific, authoritative, and positive topics that positively present the advantages of the COVID-19 vaccine, and frames with detailed narratives, scientific arguments, diversified forms of presentations, and positions in support of the COVID-19 vaccine, positively affect the effect of vaccine popularization.

**Discussion:**

Based on the experience of COVID-19 vaccine promotion in China, the results may serve as a reference for promoting innovative vaccines and handling public health affairs around the world.

## 1 Introduction

COVID-19 is highly transmissible, placing a high strain on public health systems worldwide. Due to the lack of specific drugs, many countries have adopted defensive measures to address the COVID-19 pandemic (1), and these include vaccine development, nucleic acid testing, social distancing control, etc. Vaccination against COVID-19 is one of the most successful prevention strategies (2). At the end of 2021, Omicron, a mutated strain of COVID-19, spread widely around the world. Omicron was assessed by the World Health Organization as a very high-risk strain, further stimulating efforts by countries to ramp up COVID-19 vaccination (3). China is the most populous country in the world, and the acceptance of the COVID-19 vaccine among Chinese people is significant for world health security. COVID-19 is the first pandemic spreading widely in the age of social media, big data, and artificial intelligence (4). Many studies have shown that social media can provide new information about vaccines and may influence users’ vaccine acceptance behavior (5). In China, citizens’ information reception is highly dependent on social media, the most important of which is Weibo (due to its large number of users and its role as a public information platform) (6). In this context, it is essential for vaccine promotion and public health security to investigate how popularizing information on social media affects people’s vaccine attitudes and behaviors.

Fisher et al. (7) found that public confidence in and acceptance of COVID-19 vaccines are often in flux, requiring a clear and direct way for science educators to explain vaccine characteristics to the public. During the COVID-19 pandemic, several scholars have linked the government (8, 9), organizations (10, 11), media (8), actors (12), etc., as disseminators of health information and studied their communication activities, strategies, and effects on social media. For example, Salmon et al. (13) focused on government vaccination communication strategies for different groups. Azer and Alexander (14) identified and conceptualized patterns of public vaccination behavior, starting with the communication and interaction about vaccines between the World Health Organization and the public. In addition, scholars also paid attention to the interaction between individuals and official media (10). Previous studies have shown that different types of communicators directly affect public engagement and emotional evaluation. For example, the public is more likely to trust weibos published by healthcare professionals than health media (15). Therefore, based on previous research and the current social media in China, public health experts, medical workers, government, media, health organizations, and individuals were selected as the subjects of vaccine information dissemination.

On social media platforms, the issue is an essential aspect of shaping public conversations about vaccination, providing context for what to highlight, emphasize, and exclude as a strategy for health communication (16). Harris (17) studied the theme of widespread science reporting from three stages: before, during, and after the crisis. Weaver et al. (18) proposed four themes of progress, regulation, conflict, and risk, which inspired the thematic classification of science communication. Mutua and Oloo (19) conducted content analysis and concluded that the main framework of global media on COVID-19 vaccination is conflict and responsibility, which can influence whether netizens support or reject vaccines. Some studies related to issue showed that in news communication, the different choices of issues will affect the public’s views and attitudes toward news events (20, 21), and the more relevant an issue is to users (22), the more it can promote their positive views. Therefore, the study of issues in vaccine transmission plays a vital role in how disseminators, including journalists and governments, use different issues to form and disseminate content that promotes the public’s positive attitude toward vaccine evaluation. Based on existing academic research and Weibo’s dissemination, the sample issues in this study are divided into progress, regulation, science, international news, and risk (23).

Most studies discussed vaccines on social platforms during pandemics and categorized them by topic (24–26). Vaccine information on social media can be divided into two major topics: pro-vaccine and anti-vaccine communication (27). Different issues may receive different degrees of emphasis in the popular science content of social media (28), and the choice of popular science topics will also affect the public’s acceptance of vaccines. The issues supporting vaccines are divided into the availability of vaccines, the role of vaccines in life, the increase in pandemic cases, etc. The more information about the advantages of vaccines that is disseminated, the more people can vigorously promote vaccination (29). Most anti-vaccine studies focused on vaccine hesitancy, vaccine misinformation, and the use of risk transmission. We divide the topics into five categories in the coding table: side effects, infection control effects, vaccine development, vaccine promotion, and vaccination rates.

The frame is an essential aspect in forming the public topic of vaccination. In vaccine science, it refers to highlighting, emphasizing, and excluding specific vaccine information through appropriate expressions. The vaccine science popularization frame is essential in media content analysis (30, 31). How to share health-related behaviors and participate in topic discussions is an essential theme for media content analysis in academia (31), and framing analysis as a way to organize and present information on social issues and controversies enables us to understand the dynamic public sentiment toward vaccines on the internet. Narratives and statements are often placed at the center of an individual’s conversation about health-related issues (32) and become a fundamental paradigm for understanding health communication and personal health-related behavior (33). Scannell et al. (34) studied discourse persuasion techniques such as comparison, story, and narrative frames used in posts. They found that when detailed narrative statements were used as expression methods, the public tended to view vaccines more positively. Based on previous literature, four frames, namely, the informational frame, the emotional frame, the contrasting frame, and the narrative frame, are used in this study to describe the expression of vaccine transmission on Weibo (35, 36).

The spread of positive or negative emotions on social media may influence vaccine acceptance, hesitancy, and even rejection (37). Studies have shown that the experience of vaccination has become an important topic on Weibo, where the public is more likely to express concerns about vaccines after scandalous vaccine-related events (38). Negative public perceptions of vaccines and the vaccine industry expressed in social media may bring about vaccine hesitancy and the need to restore public confidence (39). Vaccine confidence was significantly associated with participants’ concerns regarding vaccine safety and efficacy, satisfaction with incident response, and perception of vaccine benefits versus vaccine risks (40). In social media, if the sentiments and opinions of vaccine promoters are examined, objections can be addressed to some extent, and vaccine confidence can be fostered (41). The classification criteria of “positive,” “negative,” and “neutral” can be used to better evaluate the communication content of social media and are widely used in research on health issues (42). For example, Lyu et al. (43) used the sentiment analysis tool of Python to classify historical posts about vaccination on Twitter as “positive,” “negative,” and “neutral.” While weibo’s content contains positive emotions such as hope, happiness, and relief, it reflects the characteristics of more audience participation, and the audience’s evaluation attitude toward vaccination in the comment section is generally more positive. To determine the views of different groups on vaccination and thus understand its impact on public opinion, this paper’s coding table also employs this classification to evaluate weibos’ positions.

In summary, content analysis was used in this paper to extract the vaccine science popularization actors and strategies (including issue, topic, frame, and position) of sample weibos, automated sentiment analysis was employed to investigate the audience’s response (emotional tendency), and hierarchical regression analysis was conducted to interpret the impact of different strategies on the audience. Specifically, this study proposes the following research hypotheses:

*H1*: The higher the scientific authority of the actor is, the more positive the emotional tendency of the audience.

*H2*: The more relevant the issue’s content is to the COVID-19 vaccine, the more positive the audience’s emotional tendency is.

*H3*: The more the topic presents the advantages of COVID-19 vaccines, the more positive the audience’s emotional inclination.

*H4*: The more comprehensive the frame’s description of COVID-19 vaccine information is, the more positive the audience’s emotional inclination.

*H5*: The more the position leans toward supporting the COVID-19 vaccine, the more positive the audience’s emotional orientation.

## 2 Materials and methods

### 2.1 Sample collection

Based on the discussion about the COVID-19 vaccine among Weibo users, weibos containing the keywords “COVID-19 vaccine popularization” and “COVID-19 vaccine” were collected from December 1st 2019 to December 31st 2022. Then, the following weibos were eliminated in the screening process: (1) weibos with most of the content (more than 80%) not related to COVID-19 vaccine and vaccine popularization; (2) weibos mainly focusing on the history or the market of the COVID-19 vaccine (e.g., there is limited information on COVID-19 popularization); (3) weibos’ contents being mostly emotions and meaningless texts; (4) weibos mainly focusing on COVID-19 vaccine advertisements instead of COVID-19 vaccine popularization. After the data screening process, 1,058 valid samples were obtained. The study does not involve human participants. The data collected in this study comes from public content on the Internet and does not require ethical approval under Chinese law.

### 2.2 Content analysis

Based on the research hypothesis, the experience of existing studies, and sample characteristics, we analyze Actors and the weibos they posted, and construct a content-coding table of weibos’ COVID-19 vaccine science actors and strategies, including Actor, Issue, Topic, Frame, and Position. In addition, specific coding rules are presented in Table 1 to better quantify the weibo text as the unit of analysis.

**Table 1:** The dimension and indicators of coding.

Samples	Indicators	Description
Actor	Individuals	Ordinary individual users.
	Organizations	Public health organizations, hospitals, health centers, etc.
	Media	Central media, local media, media companies, etc.
	Government	The central government, local government, government departments, etc.
	Scientists	Public health experts, doctors, medical researchers, etc.
Issue	Risk	It highlights the potential risks, side effects, and complications associated with vaccination against COVID-19.
	International news	It reports on the progress of international COVID-19 vaccination and social response.
	Science	It demonstrates the scientific knowledge of the principles, characteristics, and technologies of COVID-19 vaccines.
	Regulation	It highlights the free vaccination policy of the COVID-19 vaccine and some convenient vaccination conditions.
	Progress	It introduces the progress of COVID-19 vaccination in China, including the number of people vaccinated and the vaccination rate.
Topic	Side effects	Introduces the possible adverse consequences of vaccination against COVID-19.
	Infection control effects	Introduces infection prevention rates after vaccination against COVID-19.
	Vaccine development	Introduces the development of the COVID-19 vaccine.
	Vaccine promotion	Introduces the benefits of the COVID-19 vaccine and the benefits after vaccination.
	Vaccination rates	Introduces the progress of COVID-19 vaccination in China.
Frame	Informational frame	Presents the COVID-19 vaccine information straightforwardly without complex presentation techniques.
	Emphatic frame	Highlights and emphasizes the advantages of COVID-19 vaccines based on the information provided.
	Contrasting frame	Uses contrasting narratives (e.g., comparing different vaccines, countries, populations, etc.) to highlight the benefits of COVID-19 vaccines.
	Narrative frame	Describes and demonstrates the benefits of COVID-19 vaccination in detail by providing extensive background information.
Position	Anti-vaccine	Opposes COVID-19 vaccines or COVID-19 vaccination efforts.
	Neutral	Is neutral on the COVID-19 vaccine or COVID-19 vaccination efforts.
	Pro-vaccine	Supports the COVID-19 vaccine or COVID-19 vaccination efforts.

Actor refers to the identity of the weibo publisher (44). Weibo accounts are categorized into different weibo publishers, such as government, media, and individuals. We identify and categorize Actors based on the authentication information of weibos. We follow the categorization basis of existing literature, and we identify them from the authentication information. We set five indicators, Individuals, Organizations, Media, Government, and Scientists, according to the scientific authority of the publisher (from lowest to highest) (8–11, 13).

Issue, Topic, Frame, and Position are all important indicators for measuring the strategy of popular science weibo.

Issue refers to the domain covered by weibo content (45). We set five indicators, risk, international news, science, regulation, and progress, according to the degree of relevance of issues to the COVID-19 vaccine (from low to high) (23).

Topic refers to the specific topic discussed by weibo content (36). We rate the percentage of vaccine benefits included in topics (such as safety, efficacy, accessibility, etc.) from low to high; we set five indicators, side effects, infection control effects, vaccine development, vaccine promotion, and vaccination rates (27).

Frame refers to the expression used by weibo (46). We follow the frame’s description of COVID-19 vaccine information as comprehensive (from low to high) and we set four indicators: informational frame, emphatic frame, contrasting frame, and narrative frame (35, 36).

Position refers to weibo’s position on COVID-19 (47). We set three indicators, anti-vaccine, neutral, and pro-vaccine, according to the degree of support for the COVID-19 vaccine position (from low to high) (37, 43).

According to the content analysis and coding table rules, two coders pre-coded 10% of the sample. The two coders then independently coded all valid pieces. Intercoder reliability scores were calculated using Scott’s Pi coefficient (*π*) (48). The scores all exceed 75%, indicating high coding reliability. For the same weibo or indicator, when there was a disagreement among coders, they negotiated and left the most likely coding result.

### 2.3 Sentiment analysis

When exploring the public’s emotional trend and evaluation of vaccine transmission on social media, it is necessary to build scientific and reasonable evaluation criteria. For comment sentiment recognition, we use SnowNLP, a dictionary-based Python database for Chinese sentiment analysis (49). SnowNLP has excellent short-text processing capabilities, and several studies have confirmed that it can identify emotions in weibos or weibo comments and that the accuracy rate is more than 80% (50). For this paper, all comments from a sample of 1,058 weibos, totaling 349,401, are obtained. We use SnowNLP to calculate the emotional value of each comment separately. SnowNLP automatically outputs an emotion number between 0 and 1; the closer the number is to 1, the more positive the emotion tendency is. The closer the number is to 0, the more negative the emotional direction is (51). Then, we average the sentiment values of all comments under a weibo to obtain the overall sentiment of each weibo.

### 2.4 Regression analysis

Hierarchical regression analysis can be used to detect the relationship between different dissemination subjects, dissemination strategies and dissemination effects (52). Since the Actor involves the identity of the publisher behind weibos, Issue, Topic, Frame, and Position extracted from the content of weibos can be categorized as different dissemination strategies. And different vaccine dissemination subjects and strategies bring different dissemination effects (53). Therefore, hierarchical regression is used in this paper to investigate the causal relationship between weibos’ COVID-19 vaccine popularization strategy and audience emotion. We convert all the variable values to a perfect score of 5. In the hierarchical regression analysis, we first conduct regression analysis by importing independent variable, actor and dependent variable, audience emotion. Then, we add independent variables (Issue, Topic, Frame, and Position) to conduct regression analysis and output the results.

## 3 Results

### 3.1 Actors, Weibo science popularization strategies and public emotions

Table 2 shows the number and proportion of weibos posted by every Actor. From the actor’s perspective, media (*N* = 396) has the most significant number of weibos. The leading publishers include China’s most influential mainstream media, such as People’s Daily and CCTV News, and well-known local media in China, such as The Paper and Caixin. The second largest number belongs to individuals (*N* = 358), most of whom are not identified, who participate anonymously in COVID-19 vaccine science communication and discussion. The number of Scientists (*N* = 113) comes in third. The scientists active on Weibo are mainly doctors, nurses, and other medical workers, in addition to a few influential public health experts. The number of organizations (*N* = 109) ranks fourth and their weibos are generally short and brief. The number of governments (*N* = 82) is the lowest, and the vaccine popularization information released by the Chinese government has high credibility. It can often cause a specific range of transmission and diffusion.

**Table 2:** The number and proportion of weibos posted by actors.

Samples	Indicators	Description	number	proportion
Actor	Media	Central media (e.g., @People’s Daily), Local media (eg.@Sichuan Daily), media companies (e.g., @Caixin)	396	37.43%
	Individuals	Individual user (e.g., @Chaitai CT)	358	33.84%
	Scientists	Public health experts (e.g., @Wenhong Zhang), doctor (e.g., @Doctor Weiming Luo), medical researcher (e.g., Zhuangshilihe)	113	10.68%
	Organizations	Public health organization (e.g., @Health China)	109	10.30%
	Government	Central government (e.g., @SASAC), local government (e.g., @Xian Publishing)	82	7.75%

Concerning Issue, Science (*N* = 388) has the largest number of weibos. This kind of weibos, relatively neutral, aims to reveal the characteristics of COVID-19 vaccines and provides vaccine knowledge to the public. Regulation (*N* = 262) has the second largest number of weibos, improving the convenience of COVID-19 vaccination supplied by the Chinese government and relevant departments and encouraging the public to take vaccination. The number of international news (*N* = 286) ranks third and their weibos describe the characteristics and the progress of foreign vaccines. Progress (*N* = 108), which describes progress in vaccine development and testing, ranks fourth. The number of risks (*N* = 14) is the smallest. This kind of weibos mainly informs the public about the potential risks of COVID-19, which easily induces negative emotions about vaccines.

Regarding the topic, the number of vaccine promotions (*N* = 285) is the largest. This kind of weibos persuades the public to vaccinate, emphasizing the safety and effectiveness of vaccines. The number of infection control effects (*N* = 273) is the second largest and this kind of weibos does not blindly emphasize the impact of vaccines on preventing COVID-19 infection but highlights the proportion of COVID-19 in preventing severe illness, hospitalization, and death. The number of vaccination rates (*N* = 269) ranks third. These weibos provide psychological stimulation for unvaccinated users by showing vaccination progress. Especially at the later stage of the vaccination campaign, when most people have been vaccinated against COVID-19, it is easy to stimulate the herd mentality of the unvaccinated. The content under the topic of vaccine development (*N* = 195) is mainly related to the progress issue, aiming to point out breakthroughs and progress in vaccine research and development. Side effects (*N* = 63) have the lowest number. Most weibos that describe vaccine side effects include qualifiers such as “For people with severe allergic disease, vaccination against COVID-19 may, in a small probability, cause dangerous consequences such as breathing difficulties or shock.” This emphasizes that the side effects of COVID-19 vaccines are only found in certain groups of people.

In terms of frames, the number of informational frames (*N* = 499) is the largest. Most popular science weibos still maintain the characteristics of short text and simple expression and directly list the popular science information of the COVID-19 vaccine. The number of Emphatic frames (*N* = 305) is the second, and the presentation of microblogging under this framework is similar to that of the Informational structure. Nevertheless, emphasis is given to the critical information by setting the title, adding a new line, adding a topic, and adding pictures or videos. The narrative frame (*N* = 171) ranks third. This kind of weibos is often long, with more detailed descriptions of the knowledge of the effect of the COVID-19 vaccine. Weibos involving vaccine persuasion have an argumentative process. Contrasting frames (*N* = 83) are easy to identify and often involve comparisons between domestic and foreign vaccines, and comparisons between different groups. The aim is often to persuade eligible people to vaccinate as soon as possible.

Regarding Position, the number of pro-vaccines (*N* = 786) is the largest. Most weibos are supportive of COVID-19 vaccines and vaccination efforts. Vaccination against COVID-19 is the most effective way to eliminate the COVID-19 outbreak. The number of neutral (*N* = 226) is the second largest, and this kind of weibos explains the advantages and disadvantages of vaccines and lets the audience decide whether to vaccinate or not. The number of anti-vaccines weibos (*N* = 46) is the smallest and this kind of weibos is mainly related to the safety and effectiveness of vaccines. Some weibos argue that there have been fatal cases of the COVID-19 vaccine and that vulnerable populations need to be careful. A few weibos believe that China’s domestic COVID-19 vaccine technology is backward and has poor performance in infection prevention and that it is unnecessary to vaccinate.

Regarding the emotion of the comments, most comments are neutral (*M* = 2.27, *σ* = 1.26). Positive comments usually contain positive words such as “support, belief, trust, great, like,” expressing strong trust in COVID-19 vaccines. Neutral reviews tend to be descriptive and short, making it difficult to distill public opinion about vaccines. Negative comments often contain “rejection, danger, death, allergy, fear” and can be divided into two types: concerns about vaccine safety and expressions of vaccine fear.

### 3.2 Influence of actor and Weibo science popularization strategies on public sentiment

Table 3 shows the results of the hierarchical logistic regression analysis. Model 1 examines the influence of the Actor on public sentiment. The results show that actors can explain 7.7% of the changes in public opinion, and actors positively affect the public view (OR = 0.267, *p* < 0.001). H1 is confirmed.

**Table 3:** Hierarchical regression model of actor and Weibo science popularization strategy.

	Model 1	Model 2
Actor	0.267*** (9.414)	0.199*** (6.489)
Issue		0.097* (2.503)
Topic		0.192*** (7.953)
Frame		0.062* (2.211)
Position		0.131** (3.159)
Sample size	1,058	1,058
*R* ^2^	0.077	0.142
Adjusted *R*^2^	0.077	0.138
*F*	*F* (1,1056) = 88.626, *p* = 0.000	*F* (5,1052) = 34.838, *p* = 0.000
△*R*^2^	0.077	0.065
△*F*	*F* (1,1056) = 88.626, *p* = 0.000	*F* (4,1052) = 19.812, *p* = 0.000

*p < 0.05, **p < 0.01, ***p < 0.001.

When the vaccine popularization strategy of the weibos is introduced into the model, the explanatory power of independent variables to comment sentiment is expanded, and 6.1% of the explained variance is observed. In Model 2, the actor and all strategies (Issue, Topic, Frame, Position) positively affect public sentiment. Among them are actor (OR = 0.199, *p* < 0.001), issue (OR = 0.097, *p* < 0.05), topic (OR = 0.192, *p* < 0.001), frame (OR = 0.062, *p* < 0.05), and position (OR = 0.131, *p* < 0.01). H2-5 are confirmed.

## 4 Discussion

We get 1,058 effective samples by collecting weibos related to “vaccine popularization” during the COVID-19 pandemic. Based on existing research and sample characteristics, we divide actors into Individuals, Organizations, Media, Government, and Scientists. We conduct content analysis to analyze the communication strategies from the four dimensions of Issue, Topic, Frame, and Position and measure the emotional tendency of audience comments with SnowNLP. Then, we use hierarchical regression to verify the causal relationship between vaccine popularization strategies and audiences’ emotions.

Actors can explain 7.7% of changes in public mood, and the higher the scientific authority of actors is, the more positive the audience’s emotional tendency. As the main body of information dissemination, the media posts the most, followed by individuals. At the same time, scientists (e.g., medical workers, public health experts), public health organizations, and governments publish relatively little information. Since information released by scientists, organizations, and governments is authoritative in science communication, trust in vaccine science information essentially means trust in science, scientists, and governments. As a result, data published by scientists and governments can be widely disseminated. This also inspires public health departments to appropriately increase the amount of information released by scientists, public health organizations, and governments when promoting vaccines on social media. The vaccine information published by these actors contains much professional and authoritative vaccine knowledge, which can improve the public’s understanding of vaccines. When these highly credible sources consistently present the benefits of vaccines to the public, it is easier to promote positive public attitudes toward vaccines.

The more relevant an issue is, the more positive the public’s attitude tends to be. The number of weibos of science, regulation, international news, and progress ranks the top four, meeting the audience’s needs for scientific and authoritative vaccine knowledge popularization and research and development progress, and meeting the public’s high demand for government policy directions and international vaccine situation. Moreover, when the issue is related to the personal health and vital interests of the public and has strong relevance to the public, people affected by the pandemic are more likely to believe that the information described in the communication content (54, 55) can inspire their positive emotions. The smallest number of topics is that of negative vaccine risks, which easily arouses public concerns about the safety and effectiveness of vaccines but has difficulty in encouraging the public’s inclination to vaccinate. Therefore, when disseminating international vaccine information, different actors can promote more topics of public concern and interest and promote positive public acceptance in demonstrating vaccine science and policy support and guarantees. In addition, China’s vaccine science popularizing actors should also pay attention to the accuracy of vaccine risk information, refute false statements promptly, reduce the negative impact of risk topics, and prevent mass panic caused by the spread of vaccine risk rumors (56).

It has been suggested that when a topic presents more advantages of COVID-19 vaccines, the audience’s emotional tendency is more favorable (42), which has been confirmed by the findings of this study. Vaccine promotion that emphasizes the safety and effectiveness of vaccines has the largest number of weibos, which can effectively alleviate the vaccine fear or vaccine hesitation of the audience to soothe the emotions of the audience (57, 58) so that under the topic of vaccine safety and effectiveness, a large number of users comment with positive emotions. The number of side effects is the lowest because side effects easily cause negative feelings in the audience and even cause them to resist the vaccine (42). Therefore, disseminators of vaccine information can pay more attention to the advantages of vaccines when posting weibos. When the Chinese public receives more information emphasizing the safety, effectiveness, and convenience of COVID-19 vaccines and the convenience measures provided by the government, the Chinese public’s sentiment toward vaccines is also more positive.

The more comprehensively the frame describes the COVID-19 vaccine information, the more positive the public sentiment is. Whether it is a short text, simple Informational frame, or Emphatic frame of text, pictures, or video in various forms of communication, concise or audio-visual rich media expression can attract the attention of the majority of the public to the greatest extent in the age of attention (59). The narrative frame of detailed narration and scientific argumentation is more accurately projected on audiences with a particular cultural level who are willing to learn information on social platforms to enhance users’ positive emotions toward vaccines. Contrasting frames enable the Chinese people to differentiate vaccine research progress, effects, risks, and hazards at home and abroad. The public can easily enhance positive emotions from contrasting frames highlighting vaccine advantages. Therefore, relevant actors need to describe vaccine information as comprehensively as possible and adopt more diversified forms in vaccine promotion. This suggests that when vaccine dissemination actors use multiple ways, such as pictures, videos, and texts, to disseminate vaccine information and set rich, comprehensive, and dialectical information in the content, information dissemination will be more scientific, objective, and lively, reaching audiences with different information needs to the maximum extent and helping the public understand vaccine information. This improves people’s judgment and trust in vaccines to promote vaccination.

The more the weibo’s position leans toward supporting COVID-19 vaccines, the more positive the audience’s emotional response is. A pro-vaccine approach mainly emphasizes support and trust in vaccines and is based on the belief that vaccination is an effective way to prevent and control COVID-19 (60). Kwok et al. (25) found that nearly two-thirds of all tweets on Twitter about COVID-19 vaccines were positive. Hussain et al. (61) found that the overall sentiment in vaccination-related tweets and Facebook posts was positive in the US and UK. This paper also confirms this point. Under the guidance and drive of the dissemination of content supporting vaccines, Weibo users would think that vaccines could achieve low mortality and low infectivity, meet the needs of protecting personal health, and give positive emotional evaluations such as “support” and “thumbs up.” This inspires experts, related institutions, we-media, and other communicators to pay attention to their position in vaccine promotion, provide positive information supporting vaccines, including safety and effectiveness, and at the same time, appropriately increase pro-vaccines content to convey positive emotions to the audience (62–64).

This paper verifies the feasibility of vaccine popularization on social media. Weibo is an important platform for science communication and information about vaccines can affect vaccine popularization and vaccination. This paper summarizes the advantages and strategies of China’s diverse actors for vaccine science popularization on Weibo and proposes specific suggestions for vaccine promotion. The findings elucidate the communication strategy characteristics of Chinese actors such as media, scientists, public health organizations, governments, and individuals. It offers references for other countries to effectively popularize science communication during public health crisis.

This paper has some limitations. First, the selected samples are still limited and do not include all representative samples, and such a sampling method may bias the results. Second, the categorization can still be refined. We explore the promotion strategies and effects of the COVID-19 vaccine during the period from December 1st 2019 to December 31st 2022. It needs to be further verified whether the vaccine popularization strategies and effects of each communication actor during the period of COVID-19 are effectively used for the promotion of other vaccines. In the future, we will expand further on the types of vaccines, such as focusing on influenza, HPV, and other vaccines.

## 5 Conclusion

This paper examines the COVID-19 vaccine communication activities conducted by individuals, organizations, media, government, scientists, and other diverse actors on Chinese social media (Weibo) (including communication strategies, audiences’ feedback, and communication effects). The higher the scientific authority of the actor is, the more positive the emotional tendency of the audience is. Weibos with the following communication strategies had a positive impact on the effect of vaccine popularization: scientific, authoritative, and positive issues; topics that actively presented the advantages of the COVID-19 vaccine; the frames of detailed and diversified descriptions and scientific arguments, and offers to support the position of the COVID-19 vaccine. Based on the experience of COVID-19 vaccine promotion in China, this paper proposes suggestions for vaccine promotion on social media, and the results may provide references for innovative vaccine promotion and public health affairs in countries around the world.

## Data Availability

The original contributions presented in the study are included in the article/supplementary material, further inquiries can be directed to the corresponding author.
